# A69 EXPANDED ROLE OF THE DIETITIAN IN THE CLINICAL MANAGEMENT OF PATIENTS WITH CELIAC DISEASE

**DOI:** 10.1093/jcag/gwad061.069

**Published:** 2024-02-14

**Authors:** I Asaria, J Blom, J Morgan, M Khaouli, D Armstrong, M Pinto-Sanchez

**Affiliations:** Health Sciences, McMaster University Faculty of Health Sciences, Hamilton, ON, Canada; Hamilton Health Sciences, Hamilton, ON, Canada; Hamilton Health Sciences, Hamilton, ON, Canada; Hamilton Health Sciences, Hamilton, ON, Canada; Medicine, McMaster University Faculty of Health Sciences, Hamilton, ON, Canada; Medicine, McMaster University Faculty of Health Sciences, Hamilton, ON, Canada

## Abstract

**Background:**

The only available treatment for Celiac Disease (CeD) is adhering to a strict gluten-free diet (GFD). However, a GFD has been associated with multiple nutrient imbalance and deficiencies, which are often under-recognized and remain untreated.

**Aims:**

To identify the reasons for referral to a registered dietitian (RD) and RD-recommended dietary interventions for patients with CeD attending a specialised Adult Celiac Disease Clinic.

**Methods:**

We conducted a retrospective chart review using nutrition assessment forms completed by the RD between November 2021 and May 2022. CeD diagnosis was performed through specific serology (anti-tissue transglutaminase IgA and anti-deamidated gliadin peptide IgG) and confirmed by duodenal biopsies demonstrating villous atrophy. We collected data on demographics, diagnosis date, nutritional status (BMI), micronutrients measured in serum, gastrointestinal (GI) symptoms, reason for consult, and the recommendations made by the RD. Data was collected in RedCap (v. 11.1, 2021, US) and expressed as both a proportion and Median (IQR).

**Results:**

A total of 136 visits from 102 patients were included in the analysis. Of them, 77 were female (75.5%) with a mean age of 40.3 yrs (+/- 16.0). The median year of CeD diagnosis was 4 (IQR= 6) from 2002-2022. Patients reported various symptoms including bloating (50.9%), abdominal pain (34.3%), and constipation (23.5%). 56% of patients were overweight or obese (BMI ampersand:003E25), and 4.9% were undernourished (BMIampersand:003C18). The most common nutrient deficiencies were ferritin below 30 ng/mL (58.8%), zinc below 9.4 µmol/L (23.5%), and suboptimal levels of vitamin D (38.2%). The most common reasons for referral to the RD were to assess GFD adherence (44.1%), dietary management of GI symptoms (30.4%) and nutritional optimization (24.5%). We identified 18 different interventions recommended by the RD, the most common being how to prevent gluten cross-contamination (58.8%), tips for dining out (43.1%), GF diet education (32.4%), and increasing fibre intake (30.4%). The most common reasons for follow up visits (n=34) included dietary management of GI symptoms (60.9%) and GFD adherence (43.5%).

**Conclusions:**

Patients with CeD face multiple nutritional challenges associated with following a GFD, and a number of GI symptoms. Although the GFD is the only available treatment, this review identifies the need for multiple individualized dietary interventions, which require and encourage the inclusion of a specialized RD to ensure personalized, effective care and support in the management of CeD.

**Funding Agencies:**

None

